# Drawings or 3D models: Do illustration methods matter when assessing perceived body size and body dissatisfaction?

**DOI:** 10.1371/journal.pone.0261645

**Published:** 2021-12-21

**Authors:** Cynthia Sob, Luana Giacone, Kaspar Staub, Nicole Bender, Michael Siegrist, Christina Hartmann

**Affiliations:** 1 Consumer Behavior Group, Department of Health Sciences and Technology, ETH Zurich, Zurich, Switzerland; 2 Anthropometrics & ScanLab Group, Institute of Evolutionary Medicine (IEM), University of Zurich, Zurich, Switzerland; 3 Zurich Center for Integrative Human Physiology (ZIHP), University of Zurich, Zurich, Switzerland; 4 Clinical Evolutionary Medicine Group, Institute of Evolutionary Medicine (IEM), University of Zurich, Zurich, Switzerland; Northumbria University Faculty of Health and Life Sciences, UNITED KINGDOM

## Abstract

Research has reported that both men and women experience body dissatisfaction. Among other instruments, a widely used method to assess perceived body size and body dissatisfaction are figure rating scales. Although a variety of illustration methods (e.g., three-dimensional, or 3D, models and line-drawing models) have been used to create these figure rating scales, to date, they have not been directly compared to one another. Thus, in the first study, which includes 511 participants at a mean age of 46 years old (range: 20–70), the present research work aims to assess how the line-drawing and 3D model scales, representing different body illustration methods, relate to each other. Furthermore, the first study assesses the validity of the indication of body dissatisfaction measured using these figure rating scales by comparing them to body checking or scrutinizing behavior and body appreciation levels. The project’s second study examines the two figure rating scales using objectively measured anthropometric data. In total, 239 participants at a mean age of 54 years (range: 18–94) were included. The results show that figure rating scales can be considered tools that measure perceptual body image due to their positive correlations with body checking behavior (for women) and their negative correlations with body appreciation. The 3D model and line-drawing scales show good to excellent inter-scale reliability, and both scales agree equally well with body mass index (BMI) measurements. Thus, the 3D model and line-drawing scales both seem well suited for assessing perceived body size and perceptual body dissatisfaction, suggesting that neither illustration method is superior to the other.

## Introduction

A considerable number of men and women have reported being dissatisfied with at least some parts of their bodies [1]. Body dissatisfaction is generally conceptualized as negative thoughts that a person fosters about their body [2], often in reference to body shape and size but also concerning more specific parts of the body or facial features [3]. The last few years have brought forth a selection of computerized and interactive tools to assess perceived and desired body size and shape [4–8]. Nevertheless, figure rating scales with pre-determined body illustration choices are still widely used and considered a time-saving and easy-to-administer method to assess self-ideal discrepancy as a proxy for body dissatisfaction [9, 10]. These scales are typically composed of various illustrations of the human body. Over the last few decades, a multitude of figure rating scales have emerged that use different types of body illustration methods (see references [9, 11–15] for a selection of instruments). These options prompt the question of whether one illustration method is superior to all others.

Figure rating scales assess self-reported perceived and desired body size via a set of figures that range from very thin (or very lean) to very heavy (or very muscular). In accordance with the cognitive-behavioral model of body image by Cash [3], these figure rating scales mainly tap into the construct of body image perception. This perception is formed and determined through the specific historical and developmental circumstances a person has endured and requires the ability to adequately judge one’s appearance in relation to a given reference. By calculating a self-ideal discrepancy score based on the figure ratings, it is possible to superficially infer participants’ attitudes toward their body, meaning whether they are satisfied or dissatisfied with their body size. Nevertheless, it is important to note that it is not possible to assess body image investment—meaning the emotional, behavioral, and cognitive importance of a person’s body self-evaluation—with these figure rating scales [3]. However, the discrepancy between perceived and desired body size can still be used for this purpose, and previous studies have indeed used it as an indication of body dissatisfaction [9, 13, 16].

While some figure rating scales rely on illustrations based on line drawings [14, 15, 17], some researchers have used real-life photographs of female bodies [12]. Other figure rating scales rely on three-dimensional (3D) models [11, 13], or computer-generated, biologically representative pictures, of male bodies to measure satisfaction (or dissatisfaction) with one’s body size concerning fatness and muscularity [9, 13]. For the purposes of the present study, the focus lies on the Contour Drawing Rating Scale based on line-drawing figures by Thompson and Gray [14] and 3D figures from the Body Dissatisfaction Scale by Mutale et al. [11], which are introduced in more detail below. In this study, the Contour Drawing Rating Scale [14] is referred to as the *line-drawing scale* and the Body Dissatisfaction Scale [11] as the *3D model scale*.

Body image research has used figure rating scales to assess perceptual body dissatisfaction within various study samples, ranging from college students to large-scale international population-based samples [9, 18–21]. Although these figure rating scales seem to be practical, easy-to-use, easy-to-administer, and time-saving assessments, they have faced criticism. Figure rating scales based on line-drawing figures by artists—such as the line-drawing scale [14]—have especially been criticized for appearing unrealistic and disproportionate, involving size-difference inconsistencies between the illustrated figures [22, 23]. Furthermore, the line-drawing scale has been criticized for its disproportionate arm and leg lengths and thicknesses, as well as its lack of separation between the arms and the rest of the body, which appears to be problematic for obese figures [11]. These issues might potentially make it more difficult for participants to identify themselves with one specific figure on the scale.

To address this inconsistency in body size illustrations among both male and female figure rating scales, the 3D model scale—a computer-generated figure rating scale—was developed [11]. It comprises 3D models of both men and women, using the same body measurements for either sex (e.g., the same leg and arm lengths) and metrically proportional increases in body weight and size. This scale’s measurements are based on calculated body mass index (BMI) values from 3D-generated body heights and volumes [11]. The 3D model scale might represent body sizes more adequately because its development considered BMI as an anthropometric measurement. Nevertheless, a limitation might be that the 3D models wear black clothing that could obscure important features and cues of the body that indicate adiposity or muscularity [24–26].

Although figure rating scales might lack detail compared to text-based assessment tools, especially regarding body image investment, previous research has shown that body dissatisfaction assessed with figure rating scales is associated with text-based tools that assess body image [11, 12, 16, 27]. One such tool is the Body Appreciation Scale [28], which measures appreciation and acceptance of one’s body despite internal or external negative influences. One study showed that the Body Appreciation Scale is negatively correlated with the 3D model scale for both women (*r* = -.60, *p* < .001) and men (*r* = -.46, *p* < .001) [11]. Moreover, another study found that the line-drawing scale [14] had a significantly positive correlation with a subscale of the Eating Disorder Inventory measuring body dissatisfaction among adolescent girls [16]. Thus, in agreement with previous studies, the perceived–desired body image discrepancy assessed via figure rating scales can be considered a proxy to assess body dissatisfaction [9, 11, 16].

To further demonstrate the construct validity of perceived body size as assessed through figure rating scales, the most commonly used anthropometric parameter is BMI [11–14]. Validation study results have shown that BMI positively correlates with figure rating scales, with *r* = .59–.83 for self-reported BMI [11, 12, 14] and *r* = .69–.82 for objectively assessed BMI [13, 16]. Some studies have used other anthropometric measures as well, such as waist circumference, body fat percentage, and fat-free mass [13, 17].

In summary, to date, a multitude of figure rating scales have been developed and validated; however, to the best of the researchers’ knowledge, no study has examined these figure rating scales directly with the purpose of comparing their illustration methods. This simultaneous examination of the line-drawing and 3D model scale is important because, referring to their illustration method, one scale might be preferable over the other. Furthermore, these figure rating scales have not been tested equally for men and women: such tests have often included more young female participants as a result of student convenience sampling [11, 14, 16]. Therefore, the present research work’s primary aim, through its first study, was to compare two figure rating scales that employ line drawings or 3D models as illustration methods using a population-based and age-diverse sample, and to assess their link to text-based tools that measure body image components. The line-drawing and 3D model scales were specifically selected because, on the one hand, they represent different styles of body illustration methods and, on the other hand, they were developed in different decades. This presents an interesting context in which to explore whether older scales—such as the line-drawing scale—can compete with newer scales—such as the 3D model scale. The text-based body image assessment tools were the Body Appreciation Scale [28] for both sexes, the Body Checking Questionnaire for women [29], and the Male Body Checking Questionnaire for men [30]. The two latter tools were selected because body scrutinizing or checking behavior can be used as behavioral indications of body dissatisfaction through excessive attention to body weight or shape [29, 30].

Through Study 2, this project’s second aim was to examine age-diverse participants’ perceived body size as assessed through the two figure rating scales in relation to objectively measured anthropometric data to further contribute to the figure rating scales validation literature. To this end, BMI, body fat percentage, and waist circumference were chosen as objectively measured data for comparison to the figure rating scale assessments [12], thus overcoming self-reporting bias in anthropometric data. Furthermore, BMI was selected as the anthropometric reference measure to assess its level of agreement with the figure rating scales, especially as the 3D model scale’s computer-generated models are based on calculated BMI values [11]. Moreover, to date, BMI is still considered a relevant measure to determine thresholds for elevated disease risks such as diabetes, hypertension, and other cardiovascular illnesses [31].

Thus, these two studies aimed to fill the research gap regarding the comparison of figure rating scales’ illustration methods. This overall project investigated the figure rating scales’ stance within body image research and their link to objectively measured anthropometric data to make statements about whether one illustration method is superior to the other.

## Study 1

Comparing the line-drawing scale [14] and 3D model scale [11] with one another, body checking behavior, and body appreciation is important to assess their validity. Accordingly, the two figure rating scales and their level of agreement were compared to analyze their relationship with each other. Based on previous research [11, 12] that found negative correlations between body appreciation and body dissatisfaction as measured by figure rating scales, the researchers expected to find the same negative relationship between body dissatisfaction measured using the figure rating scales and the Body Appreciation Scale for both sexes. Additionally, the researchers expected a higher frequency of body checking behavior to be positively associated with body dissatisfaction as measured with the figure rating scales.

## Methods

### Participants and procedure

The data collection took place via an online survey in May 2019. The participants were recruited in the German-speaking part of Switzerland from a commercial sampling service provider’s internet panel (Respondi AG). Quota samples were used for age and sex. Before starting with the questionnaire, participants were presented with the study information on the screen where they had to give their consent. Respondents were excluded from the study if they did not complete the survey (*n* = 25) or if their total survey duration was less than half of the median total survey duration (*n* = 26, median = 9.3 minutes). Additionally, pregnant women (*n* = 3) and participants without a clear gender classification (*n* = 1) were excluded. The study’s final sample consisted of 511 participants. The mean age of the sample was 46 years (SD = 14). The mean BMI was 25.5 kg/m^2^ (SD = 6.3) for women and 26.0 kg/m^2^ (SD = 4.4) for men. Table 1 provides further participant socio-demographic information.

**Table 1:** Demographic characteristics of Study 1’s sample.

		*n*	%
Sex (*n* = 511)	Men	245	47.9
	Women	266	52.1
Age (*n* = 511)	18–39 years	187	36.6
	40–64 years	258	50.5
	≥ 65 years	65	12.9
Education level (*n* = 511)^a^	Low	19	3.8
	Medium	246	48.1
	High	246	48.1
BMI (*n* = 511)	Underweight (< 18.5 kg/m^2^)	21	4.1
	Normal weight (18.5–24.9 kg/m^2^)	249	48.7
	Overweight (25–29.9 kg/m^2^)	154	30.2
	Obese (≥ 30 kg/m^2^)	87	17.0

BMI (body mass index) was calculated with self-reported weights and heights.

^a^ *Education level* was divided into three categories: *low* (no education, primary school, and lower secondary school), *medium* (vocational school), and *high* (higher secondary school, college, and university).

### Measures

#### Figure rating scales

**Fig 1:**
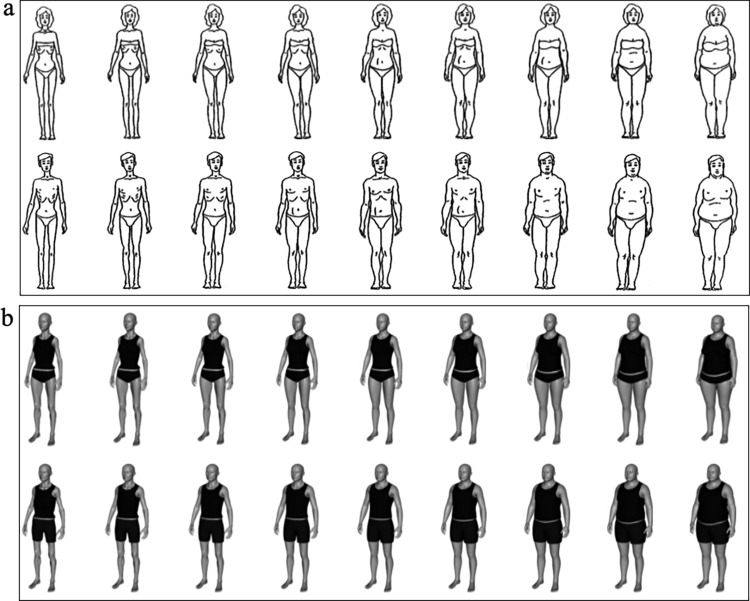
Illustrations of the used figure rating scales. (a) The line-drawing scale [14] with female figures in the first row and male figures in the second row. (b) The 3D model scale [11] with female figures in the first row and male figures in the second row. These figures are presented from left to right in ascending order, from very thin to very heavy.

Perceived body size and desired body size were assessed using two different body scales per sex. The line-drawing scale [14] and the computer-based 3D model scale [11] each included nine body figures. Both male and female versions of these scales were available (see Fig 1 for illustrations of the figure rating scales).

The participants were asked to select the body figure that best reflected their perceived body size and desired body size. They were first presented with the respective figure rating scales with the instructions for perceived body size (“Please select the illustration that looks most like you”), followed by the instructions for desired body size (“Please select the illustration you most want to look like”). All figures were presented simultaneously in ascending order from left to right, from (1) very thin to (9) very heavy. Body dissatisfaction was calculated using the difference between the participants’ perceived and desired body size. A positive score indicated that participants preferred a thinner body size, whereas a negative score indicated that they preferred a larger body size.

#### Body appreciation

To assess body appreciation level, this study utilized a German translation of the Body Appreciation Scale [28]. It features 13 items measuring four aspects of positive body image: *favorable opinions of the body*, *respect for the body*, *acceptance of the body*, and *protection of the body*. Example items include “Despite its imperfections, I still like my body” and “I do not focus a lot of energy being concerned with my body shape or weight.” One item was slightly modified to address both male and female participants (“I do not allow unrealistic images of women/men presented in the media to affect my attitudes toward my body”). The items were scored on a 5-point scale from 1 (*never*) to 5 (*always*). A mean score was then calculated from them, with higher scores indicating higher levels of body appreciation. The Cronbach’s alpha was α = .92.

#### Female body checking behavior

The German version [32] of the 23-item Body Checking Questionnaire [29] was used to assess female body checking behavior. The items were rated on a 5-point scale, ranging from 1 (*never*) to 5 (*very often*). Questionnaire items included “I touch underneath my chin to make sure I don’t have a double chin,” “I check to see how my bottom looks in the mirror,” and “I check to see if my fat jiggles.” A higher sum for these scores indicated a higher frequency of female body checking behavior. The Cronbach’s alpha was α = .93.

#### Male body checking behavior

For male participants, a German translation of the Male Body Checking Questionnaire [30] was used. It incorporated specific items formulated for men relating to muscle mass, reduced subcutaneous body fat, and the shape or feel of specific muscles. Questionnaire items included “I will check the size and shape of my muscles in most reflective surfaces” and “I ask others to feel my muscle to ensure their size or density.” The questionnaire’s 19 items were scored on a 5-point Likert scale from 1 (*never*) to 5 (*very often*). A higher sum-score indicated a higher frequency of male body checking behavior. The Cronbach’s alpha was α = .95.

#### Sociodemographic data

The participants were asked to indicate their age, sex, educational level, vocational status, height in centimeters, and weight in kilograms. The two latter data points were used to calculate self-reported BMI by dividing weight in kilograms by height in square meters (kg/m^2^).

### Statistical analyses

Outlier BMI analyses were conducted to reveal extreme BMI values. Even though severe over- or underweight body types might be indicative of underlying health issues [33], the researchers decided to include these participants within the study because these values are biologically plausible, and the figure rating scales aim to assess both under- and overweight body types. As a precaution, the participants with extremely low BMI values were temporarily removed to conduct all analyses. It was decided to not exclude them from the dataset because they did not considerably influence the results. To examine links between perceived and desired body size in the two figure rating scales, Pearson’s correlation coefficients were calculated. Agreement between the 3D model and line-drawing scales for perceived and desired body sizes were calculated with intra-class correlation (ICC) estimates and 95% confidence intervals (95% CI) based on single-rating, absolute-agreement, 2-way mixed-effects models [34, 35]. To interpret the ICC values, the 95% CI were considered [34]. To assess the validity of the body dissatisfaction measured by the figure rating scales, Pearson’s correlation coefficients were calculated with self-reported BMI, body checking behavior, and body appreciation levels; all analyses were conducted separately for men and women. Pearson correlations were compared to Spearman rank correlations, and both methods allowed the same conclusions. Therefore, Pearson correlations alone were presented. R version 4.0.2 [36] was used for these analyses with the following packages:, *psych* [37], *tidyverse* [38], *ggpubr* [39], *sjPlot* [40], and *irr* [41].

## Results

### Associations between the figure rating scales and BMI

**Fig 2:**
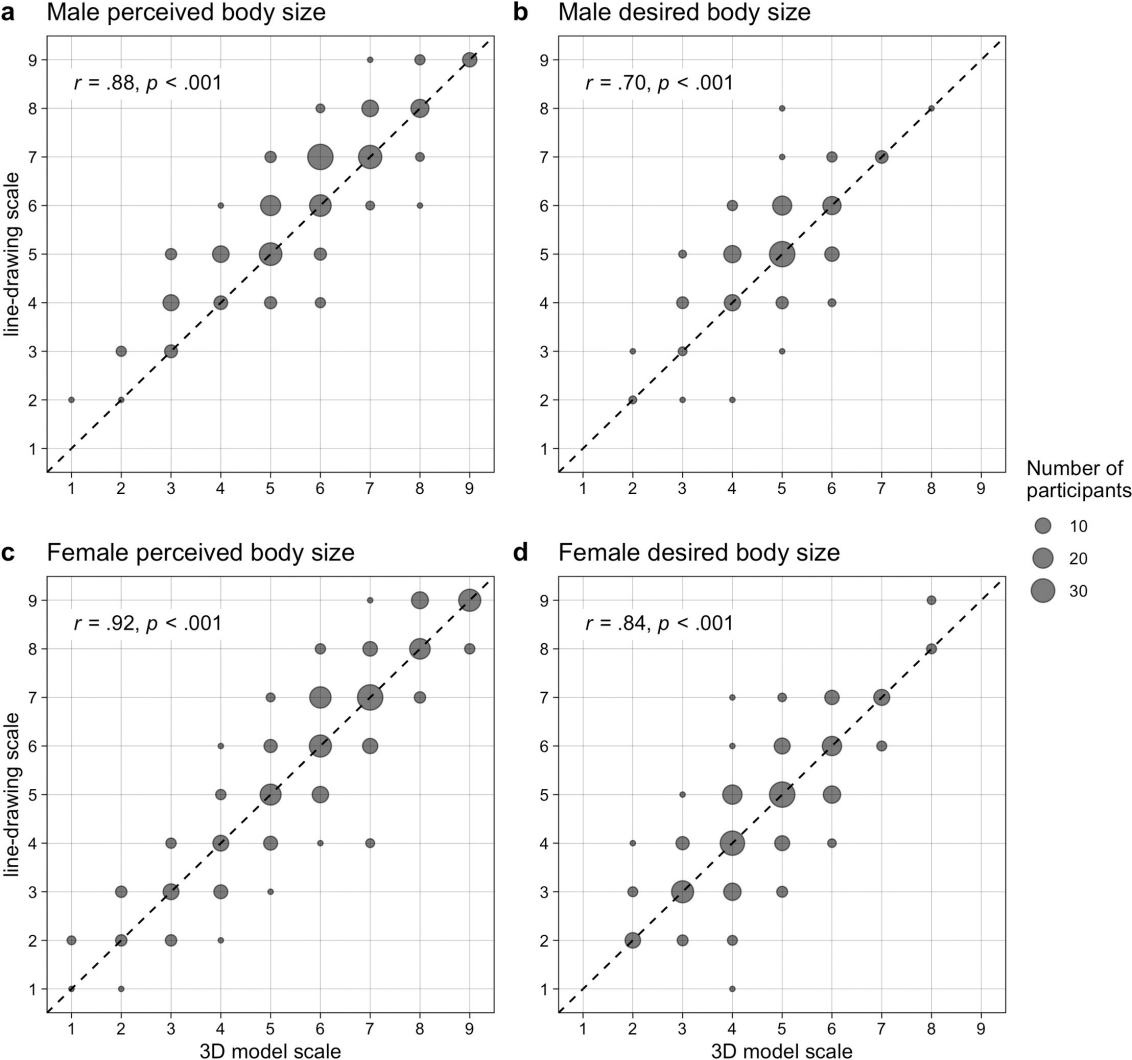
Scatterplots showing the correlations between the line-drawing scale (y-axis) and 3D model scale (x-axis). The different sizes of the grey dots represent the number of participants who chose the same figures on the figure rating scales. The dashed line represents a perfect correlation between both scales. (a) Male perceived body size; (b) male desired body size; (c) female perceived body size; (d) female desired body size.

Pearson’s correlation coefficients were calculated to assess the relationships between perceived body size and desired body size in the 3D model and line-drawing scales for both sexes. The results show that for both sexes’ perceived and desired body sizes, strong positive correlations existed between the 3D model and line-drawing scales (perceived body size—men: *r* = .88, *p* < .001; perceived body size—women: *r* = .92, *p* < .001; desired body size—men: *r* = .70, *p* < .001; desired body size—women: *r* = .84, *p* < .001). This means that both figure rating scales are indeed highly related. Fig 2 shows scatterplots illustrating these correlations. Additionally, the BMI values calculated from the participants’ self-reported weight and height show high positive correlations with perceived body size as assessed by the two figure rating scales for men (3D model scale: *r* = .73, *p* < .001; line-drawing scale: *r* = .70, *p* < .001) and women (3D model scale: *r* = .78, *p* < .001; line-drawing scale: *r* = .76, *p* < .001). Thus, self-reported BMI seems to be similarly linearly associated with both figure rating scales for men and women.

### Agreement between the figure rating scales

**Fig 3:**
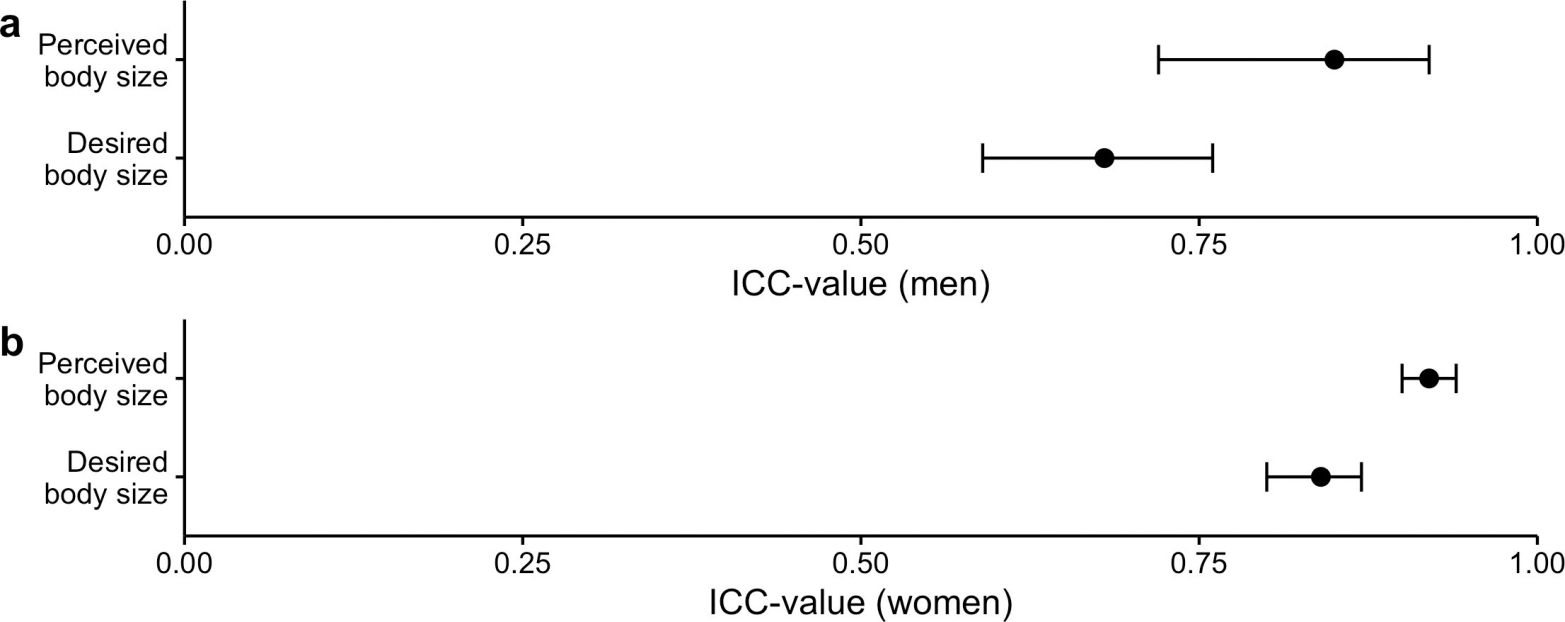
Level of agreement between figure rating scales. The intraclass correlation (ICC) values and confidence intervals (CI) for perceived body size and desired body size between the 3D model and line-drawing scales are also shown for (a) men and (b) women.

Levels of agreement between the line-drawing and 3D model scales were analyzed using ICCs and their 95% CI based on single-rating, absolute-agreement, 2-way mixed-effects models [34, 35]. Per Fig 3, the results for men show that the ICC between the 3D model and line-drawing scales for perceived body size (ICC = .85, 95% CI = [.72, .91]) indicate moderate to excellent inter-scale reliability, and the ICC for the corresponding figure rating scales for desired body size (ICC = .68, 95% CI = [.59, .76]) show moderate to good inter-scale reliability. For women, the ICC value between the 3D model and line-drawing scales for perceived body size (ICC = .92, 95% CI = [.90, .94]) indicate excellent inter-scale reliability, while the ICC for the corresponding figure rating scales for desired body size (ICC = .84, 95% CI = [.80 .87]) show good inter-scale reliability. Thus, both figure rating scales similarly demonstrate how the participants chose their perceived and desired body sizes, despite potentially involving more discrepancy between scales regarding desired body size choices for both sexes.

### Associations between body dissatisfaction measured by the figure rating scales, body appreciation, body checking behavior, BMI, and age

Separate Pearson’s correlation coefficients were calculated for the two figure rating scales, self-reported BMI, body appreciation levels, and body checking behavior for both sexes (Tables 2 and 3). For women, self-reported BMI correlates positively with body dissatisfaction measured using the two body figure scales (3D model scale: *r* = .46, *p* < .001; line-drawing scale: *r* = .47, *p* < .001). Similar results exist for men, where both scales are positively correlated with self-reported BMI (3D model scale: *r* = .54, *p* < .001; line-drawing scale: *r* = .49, *p* < .001). Thus, for both sexes, body dissatisfaction levels measured with the selected figure rating scales increase with higher self-reported BMI. Furthermore, for women, the results show medium-to-large negative correlations between body appreciation levels and body dissatisfaction measured with the two figure rating scales (3D model scale: *r* = -.43, *p* < .001; line-drawing scale: *r* = -.50, *p* < .001). For men, small negative correlations are observed between the figure rating scales and body appreciation levels (3D model scale: *r* = -.18, *p* < .001; line-drawing scale: *r* = -.20, *p* < .001). Thus, these results confirm previous findings [11, 12] of negative correlations between body appreciation and body dissatisfaction.

**Table 2:** Pearson correlation coefficients between body dissatisfaction measured with the figure rating scales (3D model scale, line-drawing scale), body appreciation levels, body checking behavior, body mass index (BMI), and age for men (n = 245).

Body dissatisfaction	M(SD)	min–max	1.	2.	3.	4.	5.	6.
1. 3D model scale	0.8(1.3)	-3–4	---					
2. Line-drawing scale	1.0(1.3)	-4–5	.80***	---				
3. Body appreciation	3.9(0.6)	1–5	-.18**	-.20**	---			
4. Male body checking	29.8(11.9)	19–75	-.15*	-.07	-.21***	---		
5. BMI (kg/m^2^)	26.0(4.4)	13.6–48.2	.54***	.49***	-.25***	-.16*	---	
6. Age (years)	47(14)	20–70	.26***	.15*	.10	-.38***	.21**	---

*p < .05

**p < .01

***p < .001.

**Table 3:** Pearson correlation coefficients between body dissatisfaction measured with the figure rating scales (3D model scale, line-drawing scale), body appreciation levels, body checking behavior, body mass index (BMI), and age for women (n = 266).

Body dissatisfaction	M(SD)	min–max	1.	2.	3.	4.	5.	6.
1. 3D model scale	1.5(1.3)	-3–6	---					
2. Line-drawing scale	1.6(1.3)	-2–5	.82***	---				
3. Body appreciation	3.7(0.8)	1.2–5	-.43***	-.50***	---			
4. Female body checking	42.0(14.1)	23–99	.24***	.28***	-.42***	---		
5. BMI (kg/m^2^)	25.5(6.3)	10.8–49.2	.46***	.47***	-.25***	-.07	---	
6. Age (years)	45(15)	20–70	.03	.01	.15*	-.26***	.19**	---

*p < .05

**p < .01

***p < .001.

Additionally, female body checking behavior positively correlates with body dissatisfaction measured with the figure rating scales for women (3D model scale: *r* = .24, *p* < .001; line-drawing scale: *r* = .28, *p* < .001). Meanwhile, for men, there is a small negative correlation between male body checking behavior and the 3D model scale (*r* = -.15, *p* < .05) and no significant correlation with the line-drawing scale (*r* = -.07, *p* > .05). Thus, while body checking behavior correlates with body size dissatisfaction measured by the figure rating scales for women, this link is inverse or not present for men.

## Discussion

The present study demonstrates the validity of two easy-to-administer and time-saving figure rating scales to assess perceived and desired body size, and its use as a proxy to assess perceptual body size dissatisfaction within an age-diverse sample.

The two figure rating scales—the 3D model and line-drawing scales—showed high positive intercorrelations for both sexes. These results applied to perceived body size and desired body size. Additionally, the level of agreement between the two figure rating scales featured good-to-excellent reliability in perceived body size and moderate-to-good reliability in desired body size, meaning both figure rating scales seem equally well suited for participants to rate their perceived body size. Slightly more variation emerged between the figure rating scales regarding desired body size choices.

In accordance with previous findings [11, 12, 16], the present study found significant negative correlations for both sexes between body dissatisfaction measured with the two figure rating scales and body appreciation. Correlations were slightly weaker for males than for females. One possible explanation could be that the first version of the Body Appreciation Scale [28] was used in the present study, which has only been validated with a female sample. Even though previous studies, such as Mutale et al. [11], have used the first version of the Body Appreciation Scale to validate their figure rating scale for both sexes, future research should consider using the Body Appreciation Scale-2 [42], which includes a revised item selection and has been validated for men as well as women.

Significant positive correlations with body checking behavior also emerged for women and inverse to no correlations for men. This finding might be explained by the Male Body Checking Questionnaire’s stronger focus on muscularity, muscle mass, and shape [30], while body size dissatisfaction assessed with the male 3D model and line-drawing scales concerns body fat distribution and thin-ideal fat-related body dissatisfaction instead. (To make the present research more concise and focused on the thin-ideal and fat-related body dissatisfaction, the researchers decided not to present the negative correlation (*r* = -.20, *p* < .01) between body dissatisfaction measured by a figure rating scale on male muscularity [13] and male body checking behavior [30]). Thus, given these significant correlations, the 3D model scale and line-drawing scale can be considered valid to address thin-ideal and fat-related perceptual body dissatisfaction, particularly for women.

Nevertheless, the present study featured a bias risk because of its reliance on self-reported data, especially concerning self-reported weight among overweight participants [43, 44]. However, recent studies have demonstrated that the correlation between self-reported weight and measured weight is sufficiently high [45] and, thus, BMI computed from self-reported weight and height represents a valid measure for both men and women [46]. Further general limitations regarding both studies are addressed later in the General Discussion section.

In conclusion, the 3D model and line-drawing scales can be used interchangeably as easy-to-administer and time-saving assessments of perceived body size and approximate perceptual body dissatisfaction.

## Study 2

A second study was conducted to overcome Study 1’s limitations concerning self-reported weight and height to calculate BMI. In addition to BMI, waist circumference and body fat percentage were selected as objectively measured anthropometric data. Thus, Study 2 aimed to investigate the links between the participants’ responses regarding perceived body size in the 3D model and line-drawing scales with measured BMI, body fat percentage, and waist circumference. Furthermore, the figure rating scales’ agreement with measured BMI were assessed. The researchers expected that both figure rating scales would positively correlate with the anthropometric measures. The analyzed level of agreement between the scales and measured BMI further elucidated whether one illustration method is superior.

## Methods

### Participants

The participants were recruited through written invitations (Swiss Food Panel 2.0 participants [47–52]) via mailing lists from science communication events and through media advertisements. To be included in the study, participants had to be at least 18 years of age and have a good understanding of the German language. The initial sample consisted of 241 participants. For the present study’s purposes, currently pregnant participants (*n* = 2) were excluded. The final sample included 239 participants. The sample’s mean age was 54 years (SD = 19). The mean BMI was 23.1 kg/m^2^ (SD = 3.1) for women and 25.5 kg/m^2^ (SD = 3.5) for men. Missing values were addressed separately within the different analyses. Table 4 shows the sample demographics.

**Table 4:** Demographic characteristics of Study 2’s sample.

		*n*	%
Sex (*n* = 239)	Men	128	53.6
	Women	111	46.4
Age (*n* = 239)	18–39 years	56	23.4
	40–64 years	91	38.1
	65–79 years	79	33.1
	≥ 80 years	13	5.4
Education level (*n* = 224)^a^^,^ ^b^	Low	5	2.2
	Medium	75	33.5
	High	144	64.3
BMI (*n* = 234) ^b^	Underweight (< 18.5 kg/m^2^)	5	2.1
	Normal weight (18.5–24.9 kg/m^2^)	145	62.0
	Overweight (25–29.9 kg/m^2^)	66	28.2
	Obese (≥ 30 kg/m^2^)	18	7.7

BMI: body mass index.

^a^ Education level was divided into three categories: *low* (no education, primary school, and lower secondary school), *medium* (vocational school), and *high* (higher secondary school, college, and university).

^b^ The number of participants differs from the total sample due to missing values.

### Procedure

The participants were given written information containing the study procedure description and their rights followed by an informed consent form, which had to be signed prior to participation. As a first step, the participants responded to an in-house paper-and-pencil questionnaire containing the 3D model and line-drawing scales, as well as demographic questions regarding other measures. Additional measures were assessed but are not part of the present study and thus not further described. Next, the participants were asked to enter an examination room, where their anthropometric measurements were taken. They were asked to change into skin-tight clothing, which they had brought themselves, or strip down to their underwear. The participants then underwent bioelectrical impedance analysis (BIA) to assess their body composition. Following these measurements, the participants put back on their regular clothing they had arrived in. This study was approved by the Ethics Committee of ETH Zurich (EK 2019-N-08).

### Measures

#### Anthropometric measurements

A medical 8-point body composition analyzer (Seca mBCA 515, Seca AG, Reinach, Switzerland) was used to determine the participants’ total body fat percentage, among other body composition factors. The device has been validated in several studies and is often used to compare body composition measures obtained through various measurement methods [53–55]. For this BIA measurement, the participants were instructed to stand barefoot on the device’s four foot electrodes and place both hands on the four hand electrodes.

To assess their body height, the participants had to stand upright in their underwear or skin-tight clothing on a standard stadiometer (Seca 274) for measurement. Height data were transferred to the body composition analyzer (Seca mBCA 515), where the participants’ weight was measured. The device then calculated the participants’ BMI.

During the BIA measurements, manual waist circumference measurements were obtained according to World Health Organization (WHO) protocol [31] with a hand-held, stretch-resistant tape with automatic retraction (Seca 201). These measurements were taken by trained and experienced research personnel at the midpoint between the lowest point of the ribcage and the highest point of the pelvis.

#### Perceived body size

To assess perceived body size, two figure scales were used: the 3D model scale [11] and the line-drawing scale [14]. The related assessment is described above in the Methods section of Study 1.

### Statistical analyses

The same outlier analyses were conducted with the anthropometric data (BMI, body fat percentage, and waist circumference). Even though one distributional BMI outlier was detected, the researchers decided not to exclude this participant from the sample for the same reasoning as in Study 1. To examine the links between perceived body size using the figure rating scales and anthropometric measurements (BMI, body fat percentage, and waist circumference), Pearson’s correlation coefficients were calculated. Agreement between the figure rating scales and measured BMI were calculated using two-way mixed-effects, absolute-agreement, single-rater intra-class correlations (ICC3, 1) [34]. To ensure comparability between objectively measured BMI and the two figure rating scales, the scores were transformed into standardized *z*-scores for the ICC analyses. All analyses were conducted separately for men and women. As Pearson product-moment and Spearman rank correlations allowed the same conclusions, Pearson’s r alone was presented. The same R version and packages were used as in Study 1.

## Results

### Associations between perceived body size assessed by the figure rating scales and anthropometric measures

**Fig 4:**
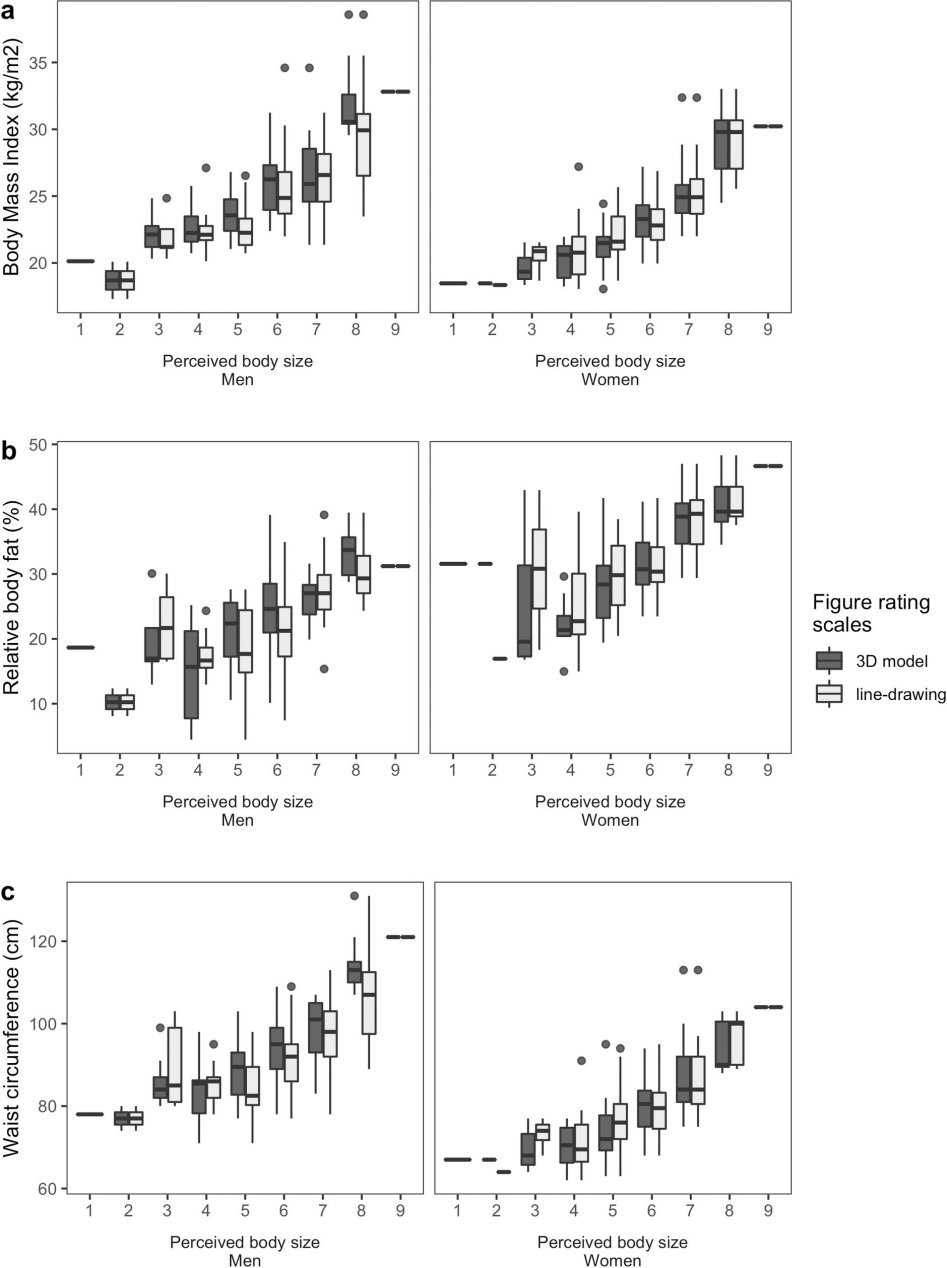
Boxplots illustrating the correlations between the figure rating scales and anthropometric measurements. The measurements of (a) body mass index (BMI), (b) relative body fat, and (c) waist circumference in centimeters are depicted by sex.

To measure the relationships between the perceived body size assessed by the 3D model scale scores (range: 1–9), line-drawing scale scores (range: 1–9), and anthropometric measures, Pearson’s correlations were calculated. The results show that both scales correlate positively with BMI, total body fat percentage, and waist circumference. High positive correlations also emerged between BMI, waist circumference, and body fat percentage and both figure rating scales for both sexes (Tables 5 and 6). Fig 4 illustrates these correlations for both figure rating scales.

**Table 5:** Descriptive statistics and Pearson correlation coefficients between perceived body size assessed with the figure rating scales and anthropometric measurements for men (n_max_ = 128, n_min_ = 122).

	M(SD)	Range	1.	2.	3.	4.	5.	6.
1. Age (years)	58(18)	20–94	---					
2. 3D model figures	5.6(1.5)	1–9	.05	---				
3. Line-drawing figures	6.1(1.4)	1–9	-.01	.83***	---			
4. BMI (kg/m^2^)	25.5(3.5)	17.3–38.6	.00	.73***	.65***	---		
5. Total body fat (%)	23.3(7.1)	4.5–39.5	.44***	.60***	.57***	.66***	---	
6. Waist circumference (cm)	93.6(11.1)	71–131	.38***	.68***	.60***	.82***	.82***	---

BMI: body mass index.

*p < .05

**p < .01

***p < .001.

**Table 6:** Descriptive statistics and Pearson correlation coefficients between perceived body size assessed with the figure rating scales and anthropometric measurements for women (n_max_ = 111, n_min_ = 107).

	M(SD)	Range	1.	2.	3.	4.	5.	6.
1. Age (years)	50(19)	18–87	---					
2. 3D model figures	5.7(1.3)	2–9	.34**	---				
3. Line-drawing figures	5.6(1.4)	1–9	.30**	.87***	---			
4. BMI (kg/m^2^)	23.1(3.1)	18.1–33.0	.34**	.77***	.71***	---		
5. Total body fat (%)	31.7(7.2)	15.0–48.3	.71***	.65***	.61***	.74***	---	
6. Waist circumference (cm)	79.7(10.2)	62–113	.53***	.70***	.67***	.87***	.81***	---

BMI: body mass index.

*p < .05

**p < .01

***p < .001.

### Agreement and comparison between perceived body size assessed by the figure rating scales and measured BMI

In accordance with Study 1’s findings, the agreement levels between the 3D model and line-drawing scales for perceived body size indicate good inter-scale reliability for men (ICC = .80, 95% CI = [.60, .89]) and good-to-excellent inter-scale reliability for women (ICC = .86, 95% CI = [.80, .91]).

**Fig 5:**
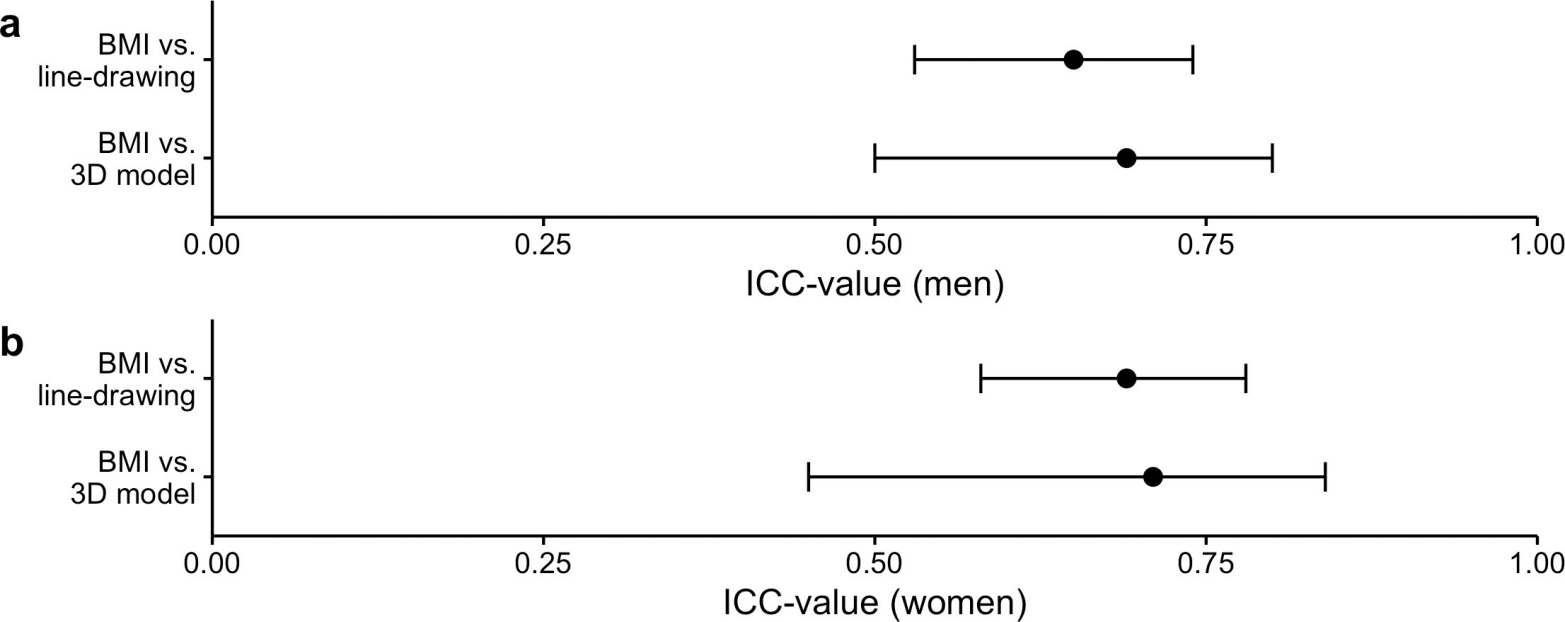
Level of agreement between body mass index (BMI) and the figure rating scales. This plot includes intraclass correlation (ICC) values and 95% confidence intervals (CI) for assessing agreement levels between BMI and the 3D model scale, as well as BMI and the line-drawing scale, for (a) men and (b) women.

Agreement for men between the 3D model scale and measured BMI shows moderate-to-good inter-measure reliability (ICC = .69, 95% CI = [.50, .80]), while the ICC between the line-drawing scale and measured BMI shows moderate inter-measure reliability (ICC = .65, 95% CI = [.53, .74]). The results for women show that the ICC between the two scales and measured BMI (3D model scale: ICC = .71, 95% CI = [.49, .84]; line-drawing scale: ICC = .69, 95% CI = [.58, .78]) indicates moderate-to-good inter-scale reliability (Fig 5). Thus, because there is an overlap of the 95% CI between the two scales for both sexes, both scales seem to depict perceived body size equally well according to BMI.

## Discussion

Study 2 showed that the 3D model scale and the line-drawing scale both highly correlate with anthropometric measurements. This finding demonstrates that both figure rating scales are equally well suited as instruments for assessing perceived body size. Consistent with the results of Study 1, Study 2 also showed that both scales feature high agreement. Furthermore, high positive correlations for both sexes emerged between both scales and BMI, body fat percentage, and waist circumference. The highest positive correlations were found for measured BMI. These findings align with previous studies that used measured and self-reported BMI as validation methods [11–13, 16, 17]. The figure rating scales were then compared in more detail to measured BMI to assess agreement levels between the measurement techniques. These results showed that both scales are equally well suited to approximate BMI measurements, with moderate-to-good inter-measurement reliability.

However, some limitations specific to Study 2 need to be addressed. First, although total body fat percentage and BMI were assessed using a precise BIA device and built-in stadiometer and scale, minor technical or calibration measurement errors might nonetheless have occurred. Because all participants were measured using the same device, and because BIA validation was not among the present study’s goals, these potential errors should not have influenced the study’s overall data quality. Second, although waist circumference measurements were obtained by trained personnel according to strict WHO protocol, marginal inter-observer differences could have occurred between measurements [56]. Finally, the last limitation is that the study’s sample size was rather small. However, similar relationships would be expected in different and larger samples.

In conclusion, both the 3D model scale and the line-drawing scale were observed to be equally well suited and valid instruments for assessing perceived body size with regard to anthropometric measurements, especially measured BMI.

## General discussion

The present research highlighted the correlations and agreement levels between figure rating scales, text-based body image instruments, and anthropometric measurements. First, the results of Study 1 showed high intercorrelations and agreement for perceived and desired body size among the 3D model and line-drawing scales for both sexes. Second, body dissatisfaction assessed with the two figure rating scales showed similar correlations with body appreciation and body checking behavior for women. For men, body checking behavior correlated negatively with the 3D model scale and not at all with the line-drawing scale. Overall, this means that both figure rating scales can possibly be used interchangeably to assess perceived and desired body size, as well as to calculate approximate perceptual body dissatisfaction. Third, the two figure rating scales proved equally well suited in representing objectively measured anthropometric data in Study 2. These results showed that the 3D model and line-drawing scales can be used interchangeably to assess perceived body size and body dissatisfaction within age-diverse, population-based study samples, with no illustration method superior to the other. These findings are important, as they emphasize that the line-drawing scale, though older and apparently less precise, does not seem significantly less adequate than the 3D model scale. This finding holds true for both sexes and across the two different samples from Studies 1 and 2.

Nevertheless, the present research has some limitations regarding both studies. First, at the individual level, the participants faced a risk of misclassifying their perceived body size due to body size misconceptions or underlying psychological conditions [57]. Still, because the incidence of such conditions in a severe form is quite small, the number of potentially concerned participants was considered small as well. Second, all figures within the figure rating scales were presented in ascending order (from very thin to very heavy) next to each other, and both figure rating scales were presented shortly after one another. This might have caused the participants to remember the location of the figure they chose on the first scale and adapt their following choices accordingly. Lastly, the findings present limited generalizability to white populations, and the wide age ranges within the samples might have influenced the results.

For future studies, it might be of interest to compare other illustration methods (e.g., realistic vs. stylized figures) and vary the presentation order of the figures to ensure more independent figure ratings between scales. It might also be of interest to compare forced choice figure rating scales with recently developed laboratory-based computerized body size estimation and dissatisfaction tasks [4–8].

In conclusion, the 3D model scale and line-drawing scales can be used interchangeably to assess perceived body size and perceptual body dissatisfaction in an easy-to-administer and time-saving way. These findings show that the body illustration method (line drawings or 3D models) within these figure rating scales might not make much of a difference for participants to indicate their perceived body size and assess body dissatisfaction. Thus, both figure rating scales could be used interchangeably in large-scale, population-based studies as quick assessments of perceived body size and approximate perceptual body dissatisfaction.
